# ALS-FTLD associated mutations of *SQSTM1* impact on Keap1-Nrf2 signalling

**DOI:** 10.1016/j.mcn.2016.08.004

**Published:** 2016-10

**Authors:** Alice Goode, Sarah Rea, Melanie Sultana, Barry Shaw, Mark S. Searle, Robert Layfield

**Affiliations:** a School of Life Sciences, University of Nottingham, UK; b Harry Perkins Institute of Medical Research, University of Western Australia, Australia; c Department of Endocrinology and Diabetes, Sir Charles Gairdner Hospital, Nedlands, Western Australia, Australia; d Centre for Biomolecular Sciences, School of Chemistry, University of Nottingham, Nottingham, UK

**Keywords:** ALS, Amyotrophic lateral sclerosis, ARE, Antioxidant response element, FTLD, Frontotemporal lobar degeneration, IKKβ, Inhibitor of nuclear factor kappa-B kinase subunit beta, Keap1, Kelch-like ECH-associated protein 1, KIR, Keap1-interacting region, LIR, LC3-interacting region, mTORC1, Mammalian target of rapamycin complex 1, *NFE2L2*, Nuclear factor erythroid 2-like 2, NQO1, NAD(P)H dehydrogenase, Quinone 1, Nrf2, Nuclear erythroid 2-related factor 2, PDB, Paget's disease of bone, RBM45, RNA binding motif protein 45, SALS, Sporadic ALS, SOD1, Superoxide dismutase 1, SQSTM1/p62, Sequestosome 1/p62 protein, TDP-43, TAR DNA-binding protein 43, UBA, Ubiquitin-associated (domain), ALS-FTLD, SQSTM1/p62, Keap1-Nrf2, Oxidative stress

## Abstract

The transcription factor Nrf2 and its repressor protein Keap1 play key roles in the regulation of antioxidant stress responses and both Keap1-Nrf2 signalling and oxidative stress have been implicated in the pathogenesis of the ALS-FTLD spectrum of neurodegenerative disorders. The Keap1-binding partner and autophagy receptor SQSTM1/p62 has also recently been linked genetically to ALS-FTLD, with some missense mutations identified in patients mapping within or close to its Keap1-interacting region (KIR, residues 347–352). Here we report the effects on protein function of four different disease associated mutations of SQSTM1/p62 which affect the KIR region. Only mutations mapping precisely to the KIR (P348L and G351A) were associated with a loss of Keap1 binding in co-immunoprecipitations comparable to wild-type SQSTM1/p62. These selective effects on Keap1 recognition were entirely rational based on protein structural models. Consistent with impaired Keap1 binding, the P348L and G351A KIR mutants showed reduced ability to activate Nrf2 signalling compared to wild-type SQSTM1/p62 in antioxidant response element (ARE)-luciferase reporter assays. The results suggest that *SQSTM1* mutations within the KIR of SQSTM1/p62 contribute to aetiology of some cases of ALS-FTLD through a mechanism involving aberrant expression or regulation of oxidative response genes.

## Highlights

• ALS-FTLD associated KIR mutations of SQSTM1/p62 disrupt Keap1 binding.
• KIR mutants of SQSTM1/p62 are unable to activate Nrf2 signalling in reporter assays.
• Some *SQSTM1* mutations may contribute to ALS-FTLD through an aberrant antioxidant stress response.

## 1 Introduction

Amyotrophic lateral sclerosis (ALS) and frontotemporal lobar degeneration (FTLD) are devastating neurological diseases that lie within the same clinicopathological spectrum, with approximately 5–10% of cases being familial. ALS is a debilitating disease characterised by loss of both upper and lower motor neurones whilst FTLD is recognised as the second most common early onset dementia. Variants in numerous genes are associated with susceptibility to ALS-FTLD including *SQSTM1* (Rubino et al., 2012), which was previously found to carry muta`tions in patients with the skeletal disorder Paget's disease of bone (PDB) (Rea et al., 2014). Coexistence of PDB and ALS-FTLD is apparently rare and precisely how different *SQSTM1* mutations (some of which are common to both disorders) can lead to either neurodegeneration or PDB is currently unknown, although in some cases co-occurrence of an additional mutation such as a pathogenic *C9orf72* expansion may account for the neurodegenerative phenotype (Almeida et al., 2015).

*SQSTM1* encodes the SQSTM1/p62 protein and the overwhelming majority of mutations associated with PDB cluster within and around the C-terminal ubiquitin-associated (UBA) domain, whereas mutations associated with ALS-FTLD occur more widely throughout the protein sequence (Rea et al., 2014, Majcher et al., 2015). In addition to the UBA domain, the multi-domain SQSTM1/p62 protein consists of an N-terminal Phox1 and Bem1p (PB1) domain, zinc finger domain (ZZ), TRAF6-binding domain, LC3-interacting region (LIR), Keap1-interacting region (KIR) and two PEST sequences. The KIR sequence allows SQSTM1/p62 to physically link to the oxidative stress-related Keap1/Nrf2 pathway (Copple et al., 2010, Lau et al., 2010), whereas the LIR allows interaction with Atg8/LC3 proteins that facilitates SQSTM1/p62's function as a receptor for ubiquitin-dependent autophagy (Pankiv et al., 2007). Due to the close proximity of the LIR and KIR, SQSTM1/p62 cannot interact with LC3 and Keap1 protein simultaneously (Jain et al., 2010). Very recently we showed that an ALS-associated L341V mutant of SQSTM1/p62, which maps directly to the LIR sequence, is defective in recognition of LC3B and associated with autophagy defects in motor neurone-like cells (Goode et al., 2016).

Previous studies have indicated that the Keap1-Nrf2 pathway, involving the transcription factor Nrf2 and its repressor protein Keap1, is altered in animal models of ALS (Mimoto et al., 2012) and post mortem tissues from patients (Sarlette et al., 2008). In addition to the prominent role of SOD1 in familial ALS, several components of the cellular oxidative stress response have been genetically linked to ALS-FTLD including variants in *NFE2L2* and *KEAP1*, encoding Nrf2 and Keap1 proteins respectively (Bergstrom et al., 2014). Furthermore Keap1 immunoreactivity has also been detected in skein-like inclusions in the spinal cords of ALS patients (Tanji et al., 2013) but the precise role of this pathway in ALS-FTLD motor neurodegeneration remains to be fully elucidated. The Keap1-Nrf2 pathway is crucial in the cellular defence response against oxidative and chemical stress (Komatsu et al., 2010). Under normal conditions monomeric Nrf2 is bound to a homodimer of Keap1 in the cytoplasm via a tight interaction with the Nrf2-ETGE motif and a weaker interaction with the Nrf2-DLGex (‘ex’ denoting ‘extended’ from the DLG tripeptide) motif (McMahon et al., 2006, Tong et al., 2006). The N-terminus of Keap1 also associates with Cullin-3 to form an E3 ubiquitin ligase complex which ubiquitinates Nrf2, signalling it to be constitutively degraded by the ubiquitin-proteasome system. Under conditions of oxidative stress Keap1 undergoes modification of cysteine residues changing its structural conformation, allowing Nrf2 to dissociate and translocate to the nucleus, where it binds antioxidant response elements (ARE) in promoter regions of many cytoprotective antioxidative genes (Kobayashi et al., 2006). Liberation of Nrf2 regulates *SQSTM1* gene expression in a positive feedback loop (Jain et al., 2010) and Nrf2 expression is also induced when SQSTM1/p62 binds to Keap1, via its KIR (residues 347–352), (Komatsu et al., 2010, Lau et al., 2010). Indeed regulation of this interaction further indicates the interdependence of Keap1-Nrf2 signalling and autophagy; SQSTM1/p62 bound to ubiquitin-modified autophagic targets is phosphorylated by mTORC1 at residue S349 within the KIR, to promote interaction with Keap1 and displacement of Nrf2 (Ichimura et al., 2013, Tanji et al., 2014). Keap1 also has a role in the autophagic degradation of IKKβ (Kim et al., 2010) which may impact NF-κB-dependent regulation of autophagy, additionally SQSTM1/p62-dependent autophagic degradation of Keap1 has been reported (Bae et al., 2013).

**Fig. 1:**
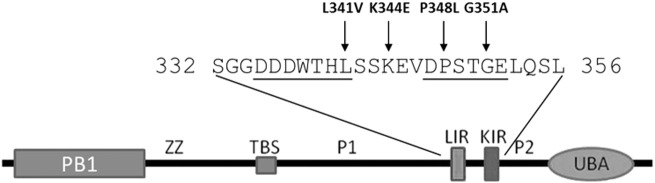
Schematic of the SQSTM1/p62 protein highlighting the amino acid sequence containing the Keap1-interacting region (KIR) and LC3-interacting region (LIR). Selected residues mutated in ALS-FTLD are indicated.

A previous study by Rubino and co-workers determining the frequency of *SQSTM1* mutations in ALS-FTLD patients attending clinics in Italy identified four novel *SQSTM1* variants, including the missense mutations K344E and P348L (Rubino et al., 2012). The K344E mutation affects a region of the protein between the LIR and KIR whereas the P348L mutation is located directly within the KIR sequence (see Fig. 1). K344E was found in 1/170 patients affected with FTLD and P348L in 1/124 patients affected with ALS (neither were present in controls). Both mutated residues are highly conserved in evolution but only P348L is predicted to be pathogenic (Rubino et al., 2012). The patient with the P348L mutation was 53 years old at age of onset but died at 55 years with a rapidly worsening condition; the FTLD patient with the K344E mutation was 69 years at age of onset and suffered from dementia. An additional disease associated KIR missense mutation, G351A, was identified in a subject from a large cohort of FTLD patients from the North West of England (Miller et al., 2015) in an individual who also had a repeat expansion in *C9orf72*, with approximately 2500 repeats (absent in controls). The patient suffered from behavioural variant FTD, with delusions as an atypical feature. She progressed slowly and died 18 years after onset of symptoms, however the authors of the study were unsure of the pathogenicity of the G351A mutation due to the co-occurrence of the *C9orf72* repeat expansion. Interestingly the G351A mutation was previously studied as an ‘artificial’ mutation to probe SQSTM1/p62-Keap1 interactions, prior to any disease association. In that context the G351A mutation was shown to reduce Keap1 binding in MBP pull-down assays and was associated with reduced Nrf2 activity in NQO1-ARE luciferase reporter assays (Jain et al., 2010). Similarly we previously found that a PDB-associated S349T KIR mutation of SQSTM1/p62, which unusually for PDB affects a region of the protein outside the UBA domain, impacts interaction with Keap1 and Nrf2 activity in reporter assays (Wright et al., 2013).

Here we verify that the disease associated G351A KIR mutation of SQSTM1/p62 impairs recognition of Keap1 and show that this mechanistic defect extends to an additional KIR mutation, P348L. In contrast, mutations located close to but not precisely mapping within the KIR (L341V, K344E) bind Keap1 normally. Consistent with this Keap1 interaction code, only the mutants associated with loss of Keap1 binding show reduced ability to activate Nrf2 signalling. Thus aberrant production of oxidative response genes may be a feature of a subset of cases of ALS-FTLD with *SQSTM1* mutations.

## 2 Materials and methods

### 2.1 Plasmids

The plasmids for expression of full-length human wild-type and L341V mutant SQSTM1/p62 protein (residues 1–440) as GST fusion proteins (pGEX-4T-1, GE Healthcare) in *E. coli* were described previously (Goode et al., 2016). The K344E, P348L and G351A mutants were created from the wild-type plasmid by site-directed mutagenesis (QuikChange kit; Stratagene) and subsequently verified by DNA sequencing. The L341V, K344E, P348L and G351A mutations were also introduced into the wild-type pcDNA3.1 His-FLAG-SQSTM1/p62 construct (Goode et al., 2014) via site-directed mutagenesis and verified by DNA sequencing. The cDNA of human LC3B was cloned into the pGEX-4T-3 plasmid (GE Healthcare) between the *Eco*RI and *SaI*I cloning sites allowing expression as a GST fusion.

### 2.2 Co-immunoprecipitations

Co-immunoprecipitations of transfected His-FLAG-SQSTM1/p62 and endogenous Keap1 have been described previously (Wright et al., 2013). Briefly, HEK293T cells were seeded in 6-well plates at 1.0 × 10^6^ cells/well. 24 h after cells were transfected with 4 μg of the wild-type or mutant His-FLAG-SQSTM1/p62 pcDNA3.1 construct (empty pcDNA3.1 in the control) for 24 h. Transfected cells were harvested and lysed in 200 μL RIPA buffer (150 mM NaCl, 1% IGEPAL® CA-630, 0.5% sodium deoxycholate, 0.1% SDS, 50 mM Tris, pH 8.0, including protease [Sigma-Aldrich, P8340] and phosphatase [Sigma-Aldrich, P5726] inhibitors [at a 1:1000 dilution]). The insoluble debris was removed by centrifugation and an aliquot of the cleared lysate (input) from each sample was kept at − 80 °C for subsequent analysis by western blot (anti-SQSTM1/p62 and anti-Keap1). Protein concentrations were determined using a BCA assay according to manufacturer's protocol and found to be highly similar for different transfections; consequently equal volumes of cleared cell lysate from each sample were used for immunoprecipitation. Cleared lysate was added to 25 μL dry volume of anti-FLAG agarose beads (Sigma) per pull-down and tumbled at 4 °C for 2 h. After incubation, the beads were pelleted and lysate removed. The beads were then washed three times and lysed in 30 μL SDS PAGE gel loading buffer. The beads were pelleted and the eluted proteins (immunoprecipitates) analysed by western blotting (anti-SQSTM1/p62 and anti-Keap1). Protein samples were separated using 5–20% SDS-PAGE gels. The separated proteins on the gel were transferred to nitrocellulose (Hybond C-Extra, Amersham Biosciences). The membrane was blocked in 5% non-fat dry milk and incubated with primary antibodies (rabbit anti-Keap1 antibody (Santa Cruz, sc-33,569: 1:1000 dilution), mouse anti-SQSTM1 antibody (BD Biosciences, 610,833: 1:5000 dilution) or mouse anti-ß-actin antibody (Sigma, A1978: 1:25,000 dilution)). Blots were then washed with TBS and incubated in the appropriate peroxidase-conjugated secondary antibody (Dako; 1:5000 dilution) and developed using Western Lightning *Plus*-ECL reagent (PerkinElmer). Three independent replicate experiments were performed and representative results are presented.

### 2.3 LC3B binding assays

LC3B was expressed and purified and pull-down assays of GST-SQSTM1/p62 proteins with immobilised LC3B were performed as described previously (Goode et al., 2016). Briefly, the GST-SQSTM1/p62 fusion proteins were expressed in 10 mL cultures of *E. coli* prior to cell lysis by sonication in 1 mL TBS-T buffer (10 mM Tris, 150 mM NaCl, 0.1% v/v Triton-× 100, pH 7.5). The lysed cells were centrifuged at 13,000 rpm for 10 min at room temperature and the cleared supernatants were diluted 1:10 in TBS-T buffer. One milliliter of each diluted lysate was incubated at 37 °C with excess glutathione-Sepharose (GE Healthcare), LC3B-Sepharose (1 mg/mL LC3B immobilised on CNBr-activated Sepharose 4B) or control-Sepharose (CNBr-activated Sepharose prepared without LC3B). The unbound proteins were then removed and the beads washed with 3 × 1 mL TBS-T buffer at 37 °C. Bound proteins were eluted from the beads with 50 μL of SDS PAGE loading buffer. Bound proteins were revealed by western blotting with the mouse anti-SQSTM1 antibody (1:5000 dilution). Each assay was repeated on a minimum of three independent occasions and representative examples are presented.

#### 2.3.1 NF-κB and Nrf2 reporter assays

Nrf2 and NF-**κ**B reporter assay protocols for SQSTM1/p62 sequences have been previously described (Rea et al., 2009, Wright et al., 2013). Briefly, for NF-**κ**B reporter assays, HEK293T cells were seeded in 96-well plates at 3 x10^4^ cells per well. 24 h later cells were transfected with 50 ng His-FLAG-SQSTM1/p62 pcDNA3.1 construct (empty pcDNA3.1 in the control), 50 ng HA-ubiquitin (a refinement to previous protocols), 40 ng of 3κB-Luc-SV40-luciferase and 10 ng of *Renilla* luciferase reporter (Rea et al., 2009) using Lipofectamine® 2000 according to the manufacturer's instructions. 24 h post-transfection cells were processed for luciferase activities according to manufacturer's instructions, using the Dual Luciferase Assay System (Promega). Experiments were performed in triplicate and assays repeated on three independent occasions.

Like NF-**κ**B assays, Nrf2 assays were performed in 96-well plates and HEK293T cells seeded at 3 x10^4^ cells per well. 24 h later cells were transfected with 100 ng His-FLAG-SQSTM1/p62 pcDNA3.1 construct, 40 ng of NQO1-ARE Firefly luciferase reporter (Zhang and Hannink, 2003) and 10 ng of *Renilla* luciferase reporter (Rea et al., 2009). As a positive control, wells were transfected with a Nrf2 expression vector in place of the SQSTM1/p62 expression vector and as a negative control an additional three wells were transfected with a NQO1 mutant reporter (pGLH-22 NQO1-ARE mutant luciferase) (Lau et al., 2010), empty pcDNA3.1 vector and the *Renilla* reporter. These transfections were included in each replicate experiment. 24-h post-transfection cells were processed for luciferase activities according to manufacturer's instructions, using the Dual Luciferase Assay System (Promega) with a GloMax-96 Microplate Luminometer (Promega). Experiments were performed in quadruplicate and assays repeated on three independent occasions.

For both NF-**κ**B and Nrf2 reporter data Firefly luciferase activity was normalised to *Renilla* luciferase activity within the same transfection and relative luciferase activity was expressed with the value for wild type SQSTM1/p62 set at 1.0. For each experiment the normalised values of the replicates were averaged, and data are presented as the mean of the average values from the separate experiments ± SEM. Statistical analyses were performed using a one-way ANOVA (GraphPad Prism6) to determine the level of significance of differences between values for wild-type SQSTM1/p62 and ALS-FTLD mutants, with significance set at *p* < 0.05.

#### 2.3.2 Molecular modelling

The effects of mutations on the structure of the Keap1-KIR complex were modelled using the molecular graphics software Pymol using the ‘mutagenesis’ tool within the ‘wizard’ menu. (The PyMOL Molecular Graphics System, Version 1.8 Schrödinger, LLC). The lowest energy side chain rotamers of the substituted residue side chain of the KIR were first generated and steric contacts were identified qualitatively from the interpenetration of the van der Waals surfaces of the mutated residue and protein side chains in the Keap1 binding pocket. No re-organisation of the latter was considered, although it is likely that intrinsic protein conformational flexibility and dynamics will partially offset some these steric contacts and result in a degree of induced fit.

## 3 Results

### 3.1 Effects of disease associated mutations on the Keap1 and LC3B binding function of SQSTM1/p62

**Fig. 2:**
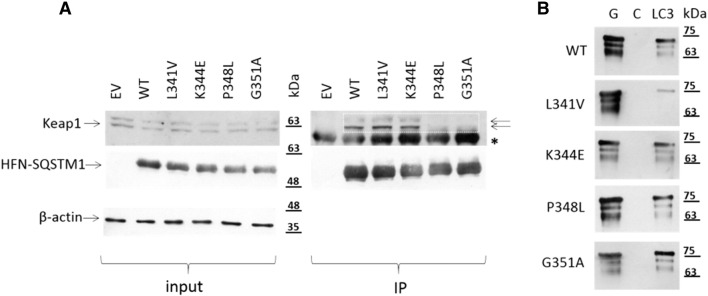
Impact of ALS-FTLD mutations on protein-protein interactions of SQSTM1/p62. (A) Effects of the ALS-FTLD mutations on SQSTM1/p62's Keap1-binding function. HEK293Ts were transfected with His-FLAG-SQSTM1/p62 (HFN-SQSTM1) pcDNA3.1 vectors as indicated or empty vector (EV) for 24 h. Levels of endogenous Keap1 (two arrows) and ß-actin as well as transfected HFN-SQSTM1 were determined by western blotting of extracted protein prior to immunoprecipitation (input). Immunoprecipitates (IPs) generated using anti-FLAG beads were blotted for bound HFN-SQSTM1 and co-bound endogenous Keap1. A representative blot of three independent experiments is presented. Boxes highlight the reduced levels of co-precipitating Keap1 with P348L and G351A mutant compared to wild-type sequence. *denotes non-specific bands. (B) The KIR mutations (P348L and G351A) and the K344E mutation do not affect SQSTM1/p62's LC3B-binding function in vitro. Indicated mutations of the full-length GST-SQSTM1/p62 protein were used in LC3B pull-down assays at 37 °C. Bacterial lysates containing the GST-SQSTM1/p62 fusions were incubated with glutathione- (G), control- (C) or LC3B-Sepharose. Bound proteins were detected by western blotting with anti-SQSTM1 antibodies. The L341V mutation serves as a control having previously been shown to cause a loss of LC3B-biding function in pull-down assays (Goode et al., 2016).

As indicated in Fig. 1, different ALS-FTLD associated missense mutations of SQSTM1/p62 map precisely within the LIR (L341V), KIR (P348L, G351A) or between these two motifs (K344E). We first examined the effects of the mutations on the SQSTM1/p62-Keap1 interaction using protein co-immunoprecipitations. Epitope (His-FLAG) tagged SQSTM1/p62 proteins were transfected in to HEK293T cells and interactions with endogenous Keap1 were determined by capture with anti-FLAG, and western blotting precipitates for Keap1 (Fig. 2A). Endogenous Keap1 was detected in immunoprecipitates following transfection with wild-type SQSTM1/p62 but not the empty vector controls. The occurrence of more than one band specific to Keap1 (panel A) has been reported previously (Fourquet et al., 2010) with the upper band representing the oxidised form of Keap1 and the lower faster migrating band the reduced form of Keap1. Consistent with previous reports using MBP pull-down assays (Jain et al., 2010), the G351A KIR mutant was largely unable to capture endogenous Keap1; further, we found that the P348L KIR mutant was also defective in Keap1 binding. A densitometric analysis of the blot presented in Fig. 2A indicates that the normalised Keap1 band intensities in the P348L and G351A IP lanes are in the order of only ~ 10% of the intensities for wild-type (data not shown). However although located only three residues away from the core KIR sequence the K344E mutation did not affect Keap1 binding of SQSTM1/p62 and likewise the more distant L341V mutant, located within the LIR, bound Keap1 normally. We also assessed the effects of the four mutations on binding to LC3B, in this case using pull-downs with purified LC3B protein on beads to capture recombinant GST-tagged SQSTM1/p62 proteins, as previously described (Goode et al., 2016). A representative western blot is shown of SQSTM1/p62 in panel B; multiple bands specific to SQSTM1/p62 were observed with the lower molecular weight bands presumably represent degradation products, resulting from adventitious bacterial proteolysis. Once again a strict interaction code was observed, with only the L341V LIR mutation affecting LC3B recognition as we previously reported (Goode et al., 2016); the K344E, P348L and G351A mutants all bound LC3B comparable to wild-type sequence (Fig. 2B), based on subjectively visual assessment of blots. Thus, different missense mutations within or around the KIR/LIR region of SQSTM1/p62 precisely impact on only the protein-protein interaction mediated by the affected motif.

### 3.2 Rationalisation of effects of the P348L and G351A mutations on the SQSTM1/p62-Keap1 interaction

**Fig. 3:**
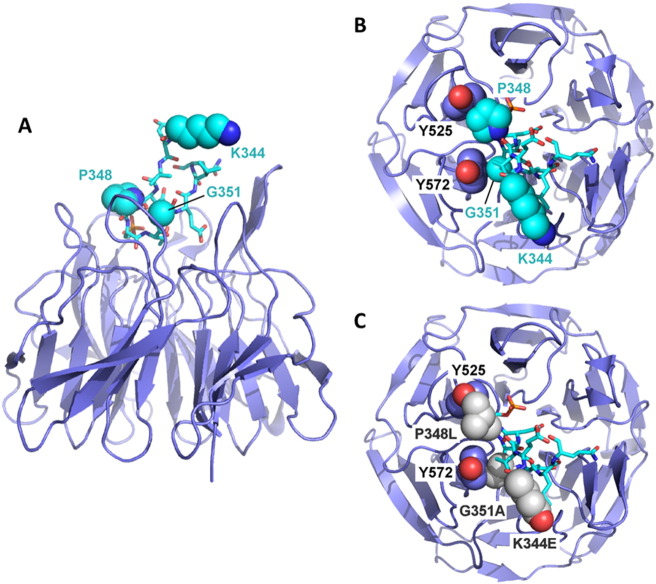
Rationalisation of effects of ALS-FTLD mutations on Keap1 binding by SQSTM1/p62. (A) Structure of Keap1 (pdb code: 3wdz) showing binding of the Keap1 interacting region (KIR) of SQSTM1/p62 (lighter blue). The KIR folds into a β-turn motif with P348 and G351 inserted into the surface binding cleft, whereas K344 remains largely solvent exposed. (B) Top-down view showing specific van der Waals contacts between the P348 and G351 residues of the KIR with Y525 and Y572, respectively, of Keap1. (C) Same structure as in (B) but showing the ALS mutations K344E, P348L and G351A in which the latter two substitutions now result in steric clashes with Y525 and Y572 of Keap1, however, there are no contacts affected by the K344E substitution.

We next sought to use structural models to rationalise how the P348L and G351A mutations selectively exert their effects on the SQSTM1/p62-Keap1 interaction. Inspection of the crystal structure (pdb code: 3wdz) of mouse Keap1 in complex with an S349-phosphorylated SQSTM1/p62 KIR peptide (equivalent and identical sequence to residues 344–354 of the human KIR) (Ichimura et al., 2013) revealed that the KIR folds into a β-turn motif with P348 and G351 both inserted into the surface binding cleft (Fig. 3). The P348L mutation causes a steric clash due to the larger Leu side chain being less readily accommodated than Pro in side chain packing with Y525 of Keap1. Furthermore the G351 residue forms close van der Waals contacts with the aromatic face of the side chain of Y572 of Keap1. Consequently, substituting Gly for Ala introduces a steric clash with the additional methyl group of the Ala, which rationalises the effects on the binding affinity. In contrast K344 of the KIR is largely solvent exposed (Fig. 3) and does not participate in contacts with Keap1 that would be affected by this substitution, entirely consistent with normal Keap1 binding of the K344E mutant in co-immunoprecipitations (Fig. 2A). Likewise, L341 is located upstream of the KIR sequence, and within the LIR (not shown in Fig. 3).

### 3.3 Effects of disease associated mutations on SQSTM1/p62's ability to regulate NF-κB and Nrf2 signalling

**Fig. 4:**
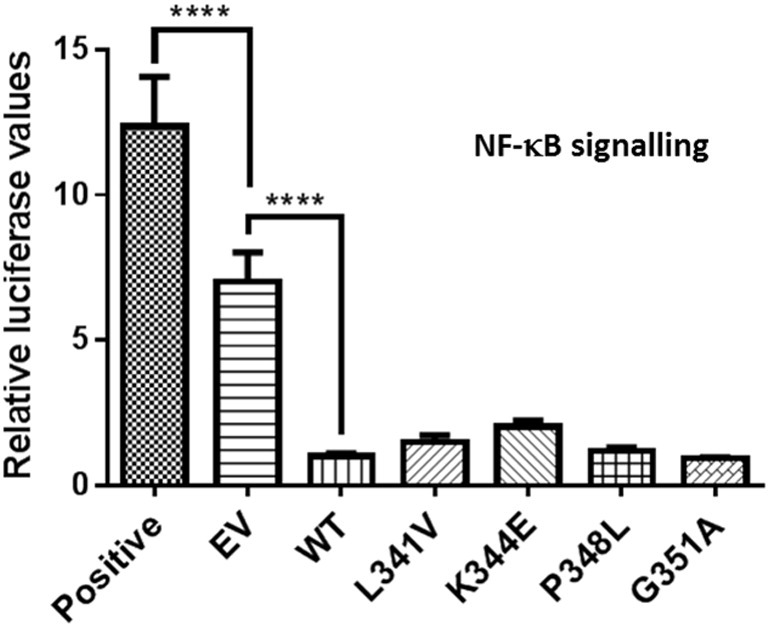
Effects of ALS-FTLD mutations on SQSTM1/p62-mediated NF-κB signalling. HEK293T cells were co-transfected with His-FLAG-SQSTM1/p62 pcDNA3.1 vectors as indicated or empty vector (EV) pcDNA3.1 control, along with an NF-κB Firefly luciferase reporter and control *Renilla* luciferase construct. Cells were harvested 24 h after transfection and luciferase activity was measured, with Firefly luciferase values normalised to *Renilla* values. Data are presented as mean values ± SEM with asterisks indicating significant differences between indicated groups. Values for the ALS-FTLD mutants were not significantly different to wild-type (WT). Positive indicates values for E396X control mutation.

Previously we reported that all but one of the many PDB-associated mutation of *SQSTM1* tested affect SQSTM1/p62's ability to regulate NF-*κ*B signalling, specifically manifesting as an activation of basal NF-*κ*B activity relative to wild-type in luciferase reporter assays in HEK293T cells (Najat et al., 2009, Goode et al., 2014) which may be related to disease aetiology. The only mutant found to lack this ability was S349T, which as noted previously, unusually for PDB affects a region of the protein outside the UBA domain, specifically within the KIR and, as we report here for the ALS-associated P348L/G351A mutants, impacts on the interaction of SQSTM1/p62 with Keap1 (and on Nrf2 signalling) (Wright et al., 2013). Thus, the effects of expression of L341V, K344E, P348L and G351A mutant SQSTM1/p62 on activation of basal NF-*κ*B signalling were similarly determined using luciferase reporter assays (Fig. 4). As a positive control, the PDB-associated E396X truncating mutant of SQSTM1/p62 was also included. HEK293T cells were co-transfected with His-FLAG-SQSTM1/p62 expression vector, NF-*κ*B luciferase reporter and a control *Renilla* luciferase plasmids. Comparable SQSTM1/p62 protein expression was confirmed by western blotting (data not shown). All four of the ALS-FTLD mutants failed to activate NF-*κ*B activity, relative to wild-type SQSTM1/p62, whereas the E396X positive control produced strong activation as previously reported (Goode et al., 2014). Thus, this defective NF-*κ*B signalling phenotype does not appear to be a feature of LIR/KIR mutants of SQSTM1/p62, at least in cell models.

**Fig. 5:**
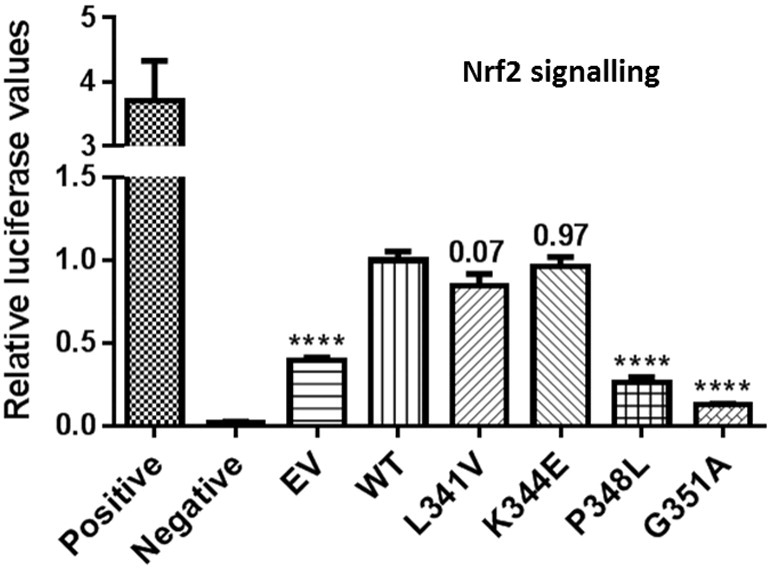
Effects of ALS-FTLD mutations on SQSTM1/p62-mediated Nrf2 signalling. HEK293T cells were co-transfected with His-FLAG-SQSTM1/p62 pcDNA3.1 vectors as indicated or empty vector (EV) pcDNA3.1 control, along with an NQO1-ARE Firefly luciferase reporter and a *Renilla* luciferase plasmid. As positive control cells were co-transfected with Nrf2 and the dual reporters. Negative controls were cells co-transfected with a mutant NQO1-ARE reporter, empty pcDNA3.1 vector, and *Renilla* luciferase plasmid. Cells were harvested 24 h after transfection and luciferase activity was measured, with Firefly luciferase values normalised to *Renilla* values. Expression of wild-type (WT) SQSTM1/p62 increased ARE-mediated luciferase activity relative to empty vector control (asterisks); expression of the P348L and G351A mutants were associated with a reduced ability to activate Nrf2 signalling, compared to WT (asterisks), whereas L341V and K344E activated Nrf2 signalling comparable to WT (*p* values indicated). Data are presented as mean values ± SEM.

Given the selective effects of the two KIR mutations (P348L, G351A) on the SQSTM1/p62-Keap1 interaction (Fig. 2A) and our previous observation that the PDB-associated S349T KIR mutant is defective in its ability to activate Nrf2 signalling (Wright et al., 2013), we assessed the effects of mutant protein expression on activation of Nrf2 signalling in reporter assays (Fig. 5). Here ARE luciferase reporter assays were employed utilising HEK293Ts co-transfected with His-FLAG-SQSTM1/p62 expression vectors, ARE luciferase reporters and a control *Renilla* luciferase plasmid as previously reported (Wright et al., 2013). As a positive control, cells were transfected with Nrf2 and the dual reporters, and as a negative control, cells were transfected with a NQO1 mutant reporter. Comparable transfected SQSTM1/p62 protein expression was confirmed by western blotting (data not shown). Expression of wild-type SQSTM1/p62 activated Nrf2 signalling relative to empty vector. Nrf2 activity associated with expression of L341V and K344E mutants was not significantly different to wild-type SQSTM1/p62. However, both the P348L and G351A KIR mutants showed significantly reduced activation of Nrf2 compared to wild-type SQSTM1/p62. Thus, the Keap1 interaction code (Fig. 2A) accurately translates to functional differences in the abilities of different SQSTM1/p62 mutants to regulate Nrf2 signalling (Fig. 5).

## 4 Discussion

In summary we show that certain ALS-FTLD associated missense mutations of SQSTM1/p62 (P348L and G351A) impair its ability to regulate Keap1-Nrf2 signalling, adding to the growing evidence that alterations in cellular defence responses against oxidative (and chemical) stress may be relevant for disease aetiology. Mechanistically, SQSTM1/p62 is able to control the cellular activity and abundance of Nrf2 through competitively binding with its inhibitory control protein Keap1 (Copple et al., 2010, Komatsu et al., 2010) and intuitively a reduced ability of P348L/G351A mutant SQSTM1/p62 to bind to Keap1 appears to manifest as a reduction in Nrf2 activity. We speculate that in the neuronal context this could, in turn, underlie an inability to mount a normal neuronal antioxidant stress response. Whether *SQSTM1* mutations in isolation are pathogenic, or whether the altered Keap1-Nrf2 signalling reported here exposes a cellular vulnerability that additional genetic and/or environmental factors combine with to promote neurodegeneration remains unclear. However, it is notable that the G351A mutation of *SQSTM1* was identified in a FTLD patient who also had a repeat expansion in *C9orf72* (Miller et al., 2015), and similarly in an FTLD-PDB family with *SQSTM1* mutation (P392L, within the UBA domain) a *C9orf72* expansion segregated with frontal cognitive impairment or dementia (but not PDB) in all but one carrier (Almeida et al., 2015). Molecular evaluation of multiple genes in ALS-FTLD cohorts, as advocated by Almeida and co-workers, is likely to be very informative in this regard.

Notably SOD1, the most widely studied protein associated with ALS-FTLD, is also connected with Nrf2 signalling. NSC-34 cells expressing G93A mutant SOD1 displayed an impairment in Nrf2 signalling with decreased numbers of shortened neurites and evidence of increased oxidative stress compared to non-mutant cells (Wang et al., 2014). Likewise in ALS mouse models expressing human SOD1 with the same mutation, activation of the Keap1-Nrf2 pathway did not properly induce the downstream cellular protective proteins in spinal motor neurons (Mimoto et al., 2012). Notably however, knockout of Nrf2 in SOD1-G93A mice had only a modest effect on disease progression, indicating that Nrf2 mediated neuroprotection is not the only protective mechanism against neurodegeneration in mice (Guo et al., 2013, Vargas et al., 2013). Further, the RNA binding protein RBM45 also modulates the antioxidant cell response through stabilising Keap1 impeding the protective antioxidant response and increased levels of RBM45 have been found in the cerebral spinal fluid of ALS patients with the protein localising to cytoplasmic inclusions in motor neurones (Bakkar et al., 2015, Li et al., 2015). This observation potentially links defects in both RNA metabolism and the Keap1-Nrf2 pathway in the pathogenesis of ALS-FTLD.

We have recently reported that the ALS-associated L341V mutation of SQSTM1/p62, which maps directly to the LIR sequence (but does not impact on Nrf2 signalling, this study), is defective in recognition of LC3B and associated with autophagy defects in motor neurone-like cells (Goode et al., 2016). Autophagy is a critical pathway for the removal of damaged and aggregation-prone proteins (including the major pathological protein TDP-43) and organelles, and is essential for neuronal survival. Whilst one interpretation of our collective findings is that there may be multiple distinct disease mechanisms in ALS-FTLD with *SQSTM1* mutations, due to the complex cross talk between signalling pathways we speculate that changes in Keap1-Nrf2 activity associated with certain mutations do have the ability to impact (converge) on autophagy. As noted previously (see Introduction) Keap1-Nrf2 signalling and autophagy intersect at various levels, and the pathways are closely interrelated. Although preliminary work using our previously established cell model (Goode et al., 2016) did not note obvious effects of the K344E or P348L mutations on incorporation of SQSTM1/p62 into acidic autophagic vesicles (data not shown) and consistent with the normal LC3B binding indicated in Fig. 2B, other models or readouts of function sensitive to altered autophagy at other stages of this multi-step process merit further investigation. It is also interesting to note that a very recent study highlighted that Nrf2 is a direct regulator of multiple autophagy genes, offering a further possible mechanism by which Nrf2 disturbances could impact on autophagy (Pajares et al., 2016).

Finally, both autophagy and the Keap1-Nrf2 pathway are strong potential therapeutic targets in neurodegeneration (Petri et al., 2012, Wang et al., 2012). Accordingly haplotypes in *NFE2L2* are associated with decreased risk and later onset of SALS respectively and one haplotype in *KEAP1* is associated with later onset of SALS (Bergstrom et al., 2014). Intriguingly, in a zebrafish model expressing mutant SOD1 the approved ALS drug riluzole and an activator of Nrf2 (apomorphine) reduced the early neuronal stress response observed in motor neurones (McGown et al., 2013) further highlighting Nrf2 signalling as an attractive potential therapeutic target (Milani et al., 2013). Indeed new pharmacological interventions in ALS-FTLD are likely to involve targeting multiple pathways, given the complex disease mechanisms which appear to be emerging.
